# Increase Crop Resilience to Heat Stress Using Omic Strategies

**DOI:** 10.3389/fpls.2022.891861

**Published:** 2022-05-17

**Authors:** Rong Zhou, Fangling Jiang, Lifei Niu, Xiaoming Song, Lu Yu, Yuwen Yang, Zhen Wu

**Affiliations:** ^1^College of Horticulture, Nanjing Agricultural University, Nanjing, China; ^2^Department of Food Science, Aarhus University, Aarhus, Denmark; ^3^College of Life Sciences, North China University of Science and Technology, Tangshan, China; ^4^Excellence and Innovation Center, Jiangsu Academy of Agricultural Sciences, Nanjing, China

**Keywords:** crop, genomics, transcriptomics, proteomics, metabolomics, phenomics, abiotic stress

## Abstract

Varieties of various crops with high resilience are urgently needed to feed the increased population in climate change conditions. Human activities and climate change have led to frequent and strong weather fluctuation, which cause various abiotic stresses to crops. The understanding of crops’ responses to abiotic stresses in different aspects including genes, RNAs, proteins, metabolites, and phenotypes can facilitate crop breeding. Using multi-omics methods, mainly genomics, transcriptomics, proteomics, metabolomics, and phenomics, to study crops’ responses to abiotic stresses will generate a better, deeper, and more comprehensive understanding. More importantly, multi-omics can provide multiple layers of information on biological data to understand plant biology, which will open windows for new opportunities to improve crop resilience and tolerance. However, the opportunities and challenges coexist. Interpretation of the multidimensional data from multi-omics and translation of the data into biological meaningful context remained a challenge. More reasonable experimental designs starting from sowing seed, cultivating the plant, and collecting and extracting samples were necessary for a multi-omics study as the first step. The normalization, transformation, and scaling of single-omics data should consider the integration of multi-omics. This review reports the current study of crops at abiotic stresses in particular heat stress using omics, which will help to accelerate crop improvement to better tolerate and adapt to climate change.

## Introduction

To achieve “zero hunger” among sustainable development goals and to ensure food security, crops must be improved especially under changeable climate conditions (Hickey et al., 2019; Janni et al., 2020). Plant scientists and breeders are under pressure and are being challenged to develop climate-smart crops with high resilience (Hickey et al., 2019). Climate change has accelerated to an unpredictable pace, which brings in extreme weather events with more frequency. For instance, extreme temperature events have hit us with stronger intensity as indicated by the maximum temperature reached, longer duration, and more frequency.

Abiotic stresses such as heat, drought, salinity, waterlogging, and particularly multiple stresses have negative effects on the world’s crop production. Recently, these negative effects have become more alarming due to accelerating global climate changes and aggravated abiotic stresses (Devireddy et al., 2021). Among the abiotic stresses, heat stress is a natural challenge for crop growth and development, which has led to significant yield loss. When the temperature is above the threshold, it can cause vegetative growth inhibition and reproductive development failure for both warm- and cool-season crops. At the molecular level, the survival strategy of crops under heat stress relies on the regulation of gene expression, resulting in the production of heat shock proteins (HSPs) (Khan and Shahwar, 2020). It is notable that HSPs play crucial roles in crops not only under heat stress but also under other stressful conditions (e.g., cold stress, water stress, and high light) by maintaining cell membrane integrity and reactive oxygen species (ROS) balance (Khan and Shahwar, 2020).

**FIGURE 1:**
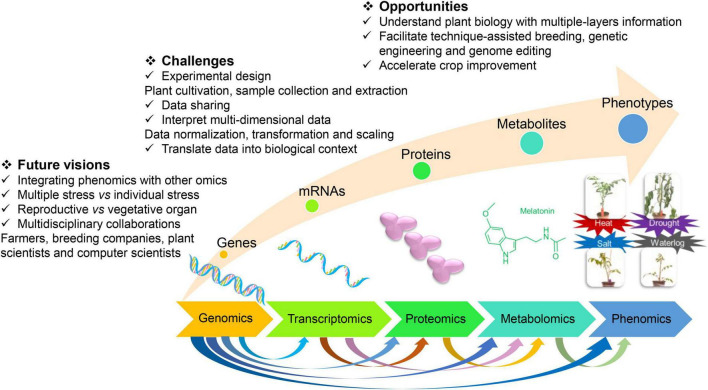
The opportunities, challenges, and future visions for crop improvement by integrating multiomics techniques.

Crop improvements can help to ease the burden to feed the 10 billion population using advanced techniques, such as genotyping, high-throughput phenotyping, and genome editing (Hickey et al., 2019). Crops respond to various abiotic stresses by activating complicated molecular networks, e.g., signal transduction, gene expressions, physiological regulation, and metabolite production. The omics techniques mainly comprise genomics, transcriptomics, proteomics, metabolomics, and phenomics (Figure 1). Single-cell omics have been widely applied to study the response of crops to abiotic stresses and investigate stress tolerance of crops, such as transcriptomics (Sharma et al., 2017; Jha et al., 2021; Wen et al., 2021). Devireddy et al. (2021) suggested that the interaction of ROS, hormones, and other signaling molecules derived the changes in metabolomics, transcriptomics, proteomics, and phenomics in plants at abiotic stresses (Devireddy et al., 2021). The integration of multi-omics is necessary to be applied to better clarify the crop tolerance and regulatory mechanism to abiotic stresses. In this review, we discussed the application of single and combined omics to elaborate crop tolerance and adaption to abiotic stresses. This review will boost the understanding of crop stress tolerance using multi-omics and benefit crop improvement.

## Omics Approaches

The major omics– techniques including genomics, transcriptomics, proteomics, metabolomics, and phenomics have enabled us to quickly, accurately, and comprehensively monitor the response of crops to environmental changes (Janni et al., 2020). The following sections provide basic knowledge of the major omics.

### Genomics

The responses of plants to abiotic stresses require regulatory changes to activate multiple genes and pathways (Bohnert et al., 2006). Next-generation sequencing techniques accelerated the progress in crop functional genomic studies (Werner, 2010; Li et al., 2018; Kersey, 2019). A bunch of genes in plants controlling key agronomic traits, especially at abiotic stresses, were identified (Ma et al., 2012; Sharma et al., 2017; Kilasi et al., 2018; Jha et al., 2021; Wen et al., 2021). Genome-wide association study (GWAS) has been widely applied to connect traits to their underlying genetics. Lots of association studies have been conducted on various crops, such as sorghum under heat stress (Chen et al., 2017), rice under drought stress (Li et al., 2017), wheat under drought stress (Bhatta et al., 2018), and wheat under salt stress (Hu et al., 2021). Li et al. (2017) conducted a GWAS using 529 rice accessions for root traits at the seed maturation stage under water deficit and found many known root-related genes being located in significant association loci (Li et al., 2017). Bhatta et al. (2018) identified 90 novel marker-trait associations (MTAs) in wheat, and 45 MTAs were in genes with a potential role in drought stress (Bhatta et al., 2018). Hu et al. (2021) investigated that 389 single-nucleotide polymorphisms (SNPs) representing 11 quantitative trait loci (QTLs) were significantly associated with yield and related traits (Hu et al., 2021).

### Transcriptomics

The RNA in plant cells consisted of messenger RNAs (mRNAs) and non-coding RNAs (ncRNAs). Transcriptomics is the study of RNA profiles including both mRNAs and ncRNAs in the cells, which has been widely applied in plant science studies in recent years (Schiessl et al., 2020). The development of high throughput sequencing enables plant scientists to study transcriptomics on a large scale (Pandit et al., 2018). In recent years, RNA-seq (RNA sequencing) using next-generation sequencing techniques has allowed the transcriptome to be more accurately characterized as compared with microarray even though qRT-PCR (quantitative real-time PCR) of selected genes is necessary to validate the sequencing results.

The expression of mRNAs in plants at various abiotic stresses was widely explored (Schiessl et al., 2020). Schiessl et al. (2020) found that winter oilseed rape (*Brassica napus*) had high regulatory diversity of drought tolerance as indicated by altered expression patterns of stress-related genes using transcriptomics (Schiessl et al., 2020). MicroRNAs (miRNAs) with approximately 20 nucleotides (nt) played roles by cleaving target mRNAs and depressing the translation of target mRNAs. Circular RNAs (circRNAs) played roles as miRNA sponges, regulators of splicing and transcription, and modifiers of parental gene expression. Long ncRNAs (lncRNAs), as key regulators in a series of essential biological processes in plants, are a group of transcripts with more than 200 nt. These ncRNAs including miRNAs, circRNAs, and lncRNAs are considered an emerging target for crop improvement (Lai et al., 2018). The global landscape of mRNAs, miRNAs, lncRNAs, and circRNAs was constructed using whole-transcriptome sequencing in plants, such as sugar beet (Li J. et al., 2020) and Chinese cabbage (Shi et al., 2021).

In recent times, it is of significant importance to study transcriptome responses of plants at the level of individual cells, since it is well known that different cell types play different biological roles in plant growth and development. Single-cell RNA-sequencing (scRNA-seq) is a high-resolution approach to study plant functional genomes and transcriptional activity of plants at the single-cell levels (Rich-Griffin et al., 2020), which helps scientists to explore heterogeneity in plants within cells types. A significant difference in some genes was detected among cell types in *Arabidopsis thaliana* at heat stress, even if the response of heat-shock proteins dominates gene expression across different cell types (Jean-Baptiste et al., 2019). Although the advanced techniques of scRNA-seq have far been widely applied in animal science, the potential in plant science has just begun to be recognized. Thereby, until now, there are few studies concerning high-throughput single-cell transcriptome in the field of plants as compared with animals.

### Proteomics

The resilience of crops to deal with various environmental changes depends on a series of alterations in their proteins as a consequence of changes in gene expression (Hakeem et al., 2012). The majority of proteins showed modification of their expression levels in crops under stress even though they were constitutively expressed in crops under normal conditions (Hakeem et al., 2012). Proteomic, the analysis of genomic complements of proteins, has been widely applied to quantify proteins and reveal protein expression changes in studying crop response to abiotic stresses (Zhao et al., 2016; Mu et al., 2017; Zhang et al., 2017; Mazzeo et al., 2018). Global determination of protein expression levels in crops at abiotic stresses was performed using isobaric tags for relative and absolute quantitation (iTRAQ), facilitating the identification of the differentially expressed proteins under control and stresses (Zhao et al., 2016). The proteome difference in crop responses to abiotic stresses could uncover protein-regulatory mechanisms and explain how specific protein functions in stress tolerance (Hakeem et al., 2012). This can be exploited in the crop breeding system to increase crop production in future challenging environments (Zhao et al., 2016).

### Metabolomics

Metabolome in the plant is comprised of primary and secondary metabolites. Primary metabolites are crucial for the synthesis of lipids, sugars, and amino acids in plants (Razzaq et al., 2019). Secondary metabolites include not only flavonoids, atropine, carotenoids, and phytic acid but also ROS, antioxidants, and coenzymes (Razzaq et al., 2019).

Metabolites such as sugars, lipids, amino acids, organic acids, and nucleotides play crucial roles in crops responding to abiotic stresses. Metabolites can link genome, transcriptome, and proteome with phenotype since metabolites are usually end products of complicated biochemical cascades (Misra et al., 2018). Metabolomics is one of the emerging methods of omics tools to probe specific metabolites and clarify the stress tolerance of crops. The relative and absolute amounts of metabolites can be determined using targeted or untargeted analyses. The analytical tools being applied for metabolomics mainly included liquid chromatography–mass spectrometry (LC-MS), capillary electrophoresis–mass spectrometry (CE-MS), gas chromatography–mass spectrometry (GS-MS), nuclear magnetic resonance (NMR), direct flow injection (DFI), high-performance liquid chromatography (HPLC), and ultra-high-performance liquid chromatography (UPLC) (Razzaq et al., 2019). The choice of tools depends on the speed, precision, and sensitivity of measurement using each tool. NMR is an efficient, non-destructive, and effective tool with high repetition. However, the NMR has a lower dynamic range, less resolution, and lower sensitivity as compared with MS. The integration of the tools, such as GC-MS and LC-MS, provided a more efficient platform for crop metabolites profiling.

### Phenomics and High-Throughput Phenotyping

Plant performance was influenced by the interaction of multiple genes and environmental factors (G × E) (Orgogozo et al., 2015). Phenomics with an automatic non-invasive sampling method, a new discipline to acquire high-dimensional phenotypic data, emerged to bridge the gap between genotype and phenotype (Egea-Cortines and Doonan, 2018). Plant phenotyping is an emerging science that connects genomics with plant agronomy and physiology, which has been considered an effective plant screening tool (Shakoor et al., 2017). However, the development of phenomics generally lagged far behind the other omics (Yang et al., 2020).

Advances in phenomics from sensors to data extraction and analysis are required to make phenomics match with other omics. Sensor techniques have enabled the recording of changes in environmental parameters and corresponding dynamic responses in crops (Yang et al., 2020). Different imaging sensors played specific roles in phenotyping facilities. First, RGB imaging can measure plant morphological characters, such as biomass and growth; however, it cannot measure physiological indexes (Yang et al., 2014). Second, fluorescent imaging can provide physiological information, such as photosynthetic ability and ROS signal (Fichman et al., 2019). Third, far-infrared thermal imaging can measure plant temperature. Fourth, hyperspectral imaging can obtain visible and near-infrared spectral and spatial information; however, it is costly with complex data processing. Fifth, the 3D imaging facilities with image-based and laser-based technologies can generate 3D models and provide spatial and volumetric traits as compared with 2D imaging. Nowadays, different imaging sensors are usually integrated into the phenomics platform to fulfill the different requirements for plant phenomic studies.

High-throughput phenotyping plays an important role in achieving the goal of understanding crop genetic architectures through combining genomic information with the whole phenotypes of main crops. Traditional crop phenotyping approaches for plant performance detection are time-consuming, labor-intensive, and mainly destructive to crops (Furbank and Tester, 2011). Accurate, efficient, non-destructive, and even dynamic high-throughput phenotyping can access crop morphology and physiology throughout plant growth and across a large population, which will benefit crop improvement. However, dealing with the large-scale phenotypic data is one of the primary bottlenecks and challenges hindering genomic studies, breeding, and improvement of crops (Yang et al., 2020), which need the continued effort from computer scientists. The efforts and cooperation from stakeholders, including plant and computer scientists, breeding companies, and farmers, are necessary to develop a joint phenotyping society (Rosenqvist et al., 2019).

## Crop Response to Abiotic Stresses Using Omics Approaches: A Case Study on Heat Stress

The genetic diversity of most crops was lost during the drive for selecting beneficial traits for human beings while breeding modern cultivars. The modern cultivars became more vulnerable to climate changes and abiotic stresses due to narrow genetic diversity. Elite cultivars with better adaption and high resilience to changeable climates were urgently needed in the breeding system to meet the growing demand for crop production (Janni et al., 2020). Conventional breeding methods, such as introgression of stress-tolerance genes from wild relatives into cultivated species, benefited crop improvement over a long period, even though it is slow and usually accompanied by the side effects of low genetic diversity (Østerberg et al., 2017). In recent times, omics techniques were applied to accelerate crop breeding and improvement to increase crop tolerance to abiotic stresses.

### Heat Stress as One of the Major Abiotic Stresses for Crop Yield Loss

Heat stresses are sensed by plants when the growth temperature is above the optimal temperature for the plants. Heat stress can lead to seed germination failure, plant growth inhibition and development retardation, and even death. Heat stress study is of great importance since it particularly negatively affects crop production more severely and frequently (Janni et al., 2020). Crops have evolved and are equipped with physiological, metabolic, and molecular regulatory mechanisms to react and adapt to the changes in surrounding temperature. However, most crops are sensitive to heat stress especially at the reproductive stage (Karwa et al., 2020). Janni et al. (2020) reported the threshold temperature for the main crops including wheat, maize, rice, chickpea, and tomato (Janni et al., 2020). More importantly, global warming has led to more frequent and more severe high temperatures, which resulted in more damage of heat stress on crops (Ohama et al., 2017).

### Heat Stress Response of Crops Using Genomics and Transcriptomics

Crop functional genomic studies have received big achievements due to the whole-genome sequencing of many crops (Kersey, 2019). Chen et al. (2017) found that 14 SNPs were directly linked to heat stress responses (HSRs) in sorghum using GWAS (Chen et al., 2017). Wen et al. (2019) identified key HSR within major QTL in tomatoes by integrating QTL mapping with RNA-seq (Wen et al., 2019). Wen et al. (2021) further analyzed key genes (*cathepsin B-like protease* 2 in tomato or *SlCathB2*) and found that the expression level of *SlCathB2* was upregulated in both heat-sensitive and heat-tolerant plants at 40°C for 12 h. Sharma et al. (2017) found three QTLs with a direct role in the heat tolerance of wheat (Sharma et al., 2017). Kilasi et al. (2018) found 213 annotated genes between boundary markers for QTL for heat stress tolerance in rice (Kilasi et al., 2018). Jha et al. (2021) identified 37 major QTLs across the genome for 12 traits, particularly heat-tolerance traits, using F_7_ RIL (recombinant inbred line) lines derived from heat-sensitive and heat-tolerant chickpea (Jha et al., 2021).

Transcript profiling offers new insights into the dynamic transcriptional changes in the plant at abiotic stresses (Bohnert et al., 2006). The transcriptomic data identified abundant genes in crops responding to high temperatures (Liao et al., 2015; Shi et al., 2017; Wen et al., 2019). For instance, the expression of 21 genes playing various roles in oxidation–reduction, metabolism, transport, transcript regulation, defense response, and photosynthetic processes were different between heat-tolerant and heat-sensitive rice at 38°C for 48 h (Liao et al., 2015). Shi et al. (2017) identified 516 upregulated and 1,261 downregulated genes in two maize types with different heat susceptibilities at 42°C for 0.5 or 3 h (Shi et al., 2017). The 4 h of 40°C induced 3,686 and 3,781 differentially expressed genes in heat-sensitive and heat-tolerant tomatoes, respectively (Wen et al., 2019). The technique of RNA-seq not only identified the heat-responsive genes but also the heat-responsive ncRNAs in crops (González-Schain et al., 2016; Mangrauthia et al., 2017; He et al., 2020; Zhou et al., 2020a,b). The ncRNAs and epigenetic regulation were shown to play crucial roles in heat-induced response in crops (Ohama et al., 2017). González-Schain et al. (2016) suggested that reproductive tissues in rice alter their transcriptome within 1 h to prevent damage caused by 38°C (González-Schain et al., 2016). The miRNAs that are only expressed in heat-tolerant rice (Nagina 22) at high temperatures were identified (Mangrauthia et al., 2017). The ncRNAs played important roles in crops being suffered to high temperatures. The expression level of 108 lncRNAs, 2,130 mRNAs, 2,477 novel circRNAs, and 348 miRNAs were significantly changed in cucumber at 42°C/32°C as compared with 28°C/18°C for 7 days (He et al., 2020). We found that the expression profiles of 74 miRNAs and 17 circRNAs were significantly altered in tomatoes at 38°C for 36 h as compared with control at 26°C (Zhou et al., 2020a,b).

### Proteomics Uncovered Responsive Proteins in Crops at Heat Stress

Proteins, as the products of genes, can directly play roles in the response of crops to heat stress. For instance, the HSPs are proteins being produced by cells under stress conditions, particularly under heat stress, in almost all organisms (Khan and Shahwar, 2020). The molecular size of the HSPs ranged from 10 to more than 100 kDa. The HSPs included HSP100, HSP90, HSP70, HSP60, and small HSP (sHSP), were distinguished based on their molecular weight (Khan and Shahwar, 2020). Heat stress transcription factors (HSFs) contributed to heat stress response in crops by regulating the expression of HSPs genes. Apart from the HSPs, a series of proteins being involved in the heat tolerance of crops were identified using proteomics (Shi et al., 2013; Zhao et al., 2016; Lu et al., 2017; Mu et al., 2017; Zhang et al., 2017; Mazzeo et al., 2018).

Upregulation of proteins for calcium signaling and hormone biosynthesis were found in heat-tolerant rice at heat stress (Shi et al., 2013). Heat stress induced degradation of ribosomal proteins in heat-sensitive rice and increase of sHSP, β-expansins, and lipid transfer proteins in heat-tolerant rice (Mu et al., 2017). Heat-tolerant and sensitive rice at heat stress exhibited 38 differentially expressed proteins, which involved signal transduction, transcript regulation, oxidation, energy metabolism, defense responses, transport, and biosynthesis (Zhang et al., 2017). The abundance of proteins involved in photosynthesis, defense, and energy metabolism increased in heat-tolerant wheat at high temperatures (Wang et al., 2015). Lu et al. (2017) found 258 heat-responsive proteins in wheat that were playing roles in redox regulation, chlorophyll synthesis, protein turnover, and carbon fixation (Lu et al., 2017). Expression levels of 135 proteins showed significant changes in maize under heat stress, and chaperone proteins and proteases played roles in the heat adaption of maize (Zhao et al., 2016). Heat stress primarily regulated the expression of proteins playing roles in assuring protein quality and ROS detoxification in tomatoes (Mazzeo et al., 2018). Thereby, proteomics has become a useful tool to analyze protein profiling and investigate the mechanisms of a plant responding to heat stress.

### Heat Stress Response of Crops Using Metabolomics

Metabolomics provides an efficient method for characterizing heat tolerance in plants, and the composition and dynamics of the metabolome in various crops at heat stress were identified using metabolomics (Chebrolu et al., 2016; Qi et al., 2016; Sun et al., 2016; Das et al., 2017; Paupière et al., 2017). Chebrolu et al. (2016) found that 11 of the top 12 metabolites with the difference between the heat-sensitive and heat-tolerant soybeans at 36°C were antioxidant compounds (Chebrolu et al., 2016). Higher accumulation of flavonoids, ascorbate precursors, and tocopherols in heat-tolerant soybean might act by alleviating ROS damage induced by heat stress in soybean (Chebrolu et al., 2016). Sun et al. (2016) suggested that biomass distribution, amino acids derived from oxaloacetate, shikimate, and its aromatic amino acid derivatives contributed to the difference in plastic response of maize to high and low temperatures (Sun et al., 2016). The biosynthesis of phytochemicals, such as daidzein, daidzin, glycitin, syringic acid, formononetin, genistein, and genistein in soybean, was affected by high temperature (Das et al., 2017). Under high temperature, 25 different metabolites (three polyols, four sugars, six organic acids, and 12 amino acids) were identified in transgenic wheat lines containing maize phosphoenolpyruvate carboxylase gene as compared with its wild type (Qi et al., 2016). Accumulation of flavonoids in the tomato microspore at short-term heat stress could play an important role in protecting pollen against heat damage (Paupière et al., 2017). Overall, metabolomics is required to complement the other omics’ knowledge and develop models for the system as a whole (Paupière et al., 2017), which can contribute to heat tolerance illumination from the aspect that the other omics cannot cover.

### Phenotyping of Heat-Tolerant Crop Genotype

Sharma et al. (2012) screened 1,274 wheat cultivars at heat stress using F_*v*_/F_*m*_ phenotyping and successfully obtain heat-tolerant and heat-sensitive types of wheat (Sharma et al., 2012). Similarly, Zhou et al. (2015) identified two heat-tolerant and two heat-sensitive tomatoes from 67 tomato genotypes using heat injury index and F_*v*_/F_*m*_ phenotyping in climate chambers (Zhou et al., 2015). More importantly, Rosenqvist et al. (2019) pinpointed that more attention to crop performance under abiotic stresses in the field condition were required (Rosenqvist et al., 2019). Poudyal et al. (2018) verified that the heat-tolerant tomato selected from climate chambers had favorable phenotyping of lower heat injury index and higher fruit yield during heat stress in the field. Heat-tolerant rice with high spikelet fertility, pollen viability as well as grain yield, and quality at high temperature was identified after phenotyping 36 rice genotypes (Karwa et al., 2020). Rascio et al. (2020) developed a low-cost phenotyping approach to analyze wheat leaf wilting after heat stress by automatically tracking leaf angle (Rascio et al., 2020). However, high-throughput plant phenomic studies have focused on the development of sensors or imaging, and high-throughput system is limited by multiple climate conditions. Specifically, the current high-throughput phenotyping platform usually cannot achieve stable high-temperature environmental conditions for heat stress treatment.

One of the most popular approaches is to screen a large scale of germplasm to identify and develop tolerant genotypes (Sharma et al., 2012; Zhou et al., 2015; Chaudhary et al., 2020; Karwa et al., 2020). The investigation of the heat-tolerant mechanism in tolerant genotypes laid the foundation for alleviating heat stress damage and improving crop resilience (Chaudhary et al., 2020; Karwa et al., 2020). The tolerant lines can be a donor for heat tolerance in crop breeding programs (Karwa et al., 2020). Meanwhile, the contrasting lines with different heat sensitivities can be crossed and segregants with desirable agronomic traits can be chosen to produce a new heat-tolerant cultivar. More importantly, breeders focus on yield-related traits at high temperature; thereby, heat-tolerant lines or cultivars with favorable production at high temperatures will be selected. This is quite important for food security worldwide, especially under the current dramatic climate change conditions together with global warming and continuously increasing population.

## Application of Multi-Omics Approaches in Crop Study at Abiotic Stresses

A multi-omics study on the stress-responsive behavior of crops examines the genes, mRNAs, proteins, metabolites, and phenotypes, which brought both opportunities and challenges (Figure 1). Biology has been revolutionized by access to large-scale datasets from genomics, transcriptomics, proteomics, metabolomics, and phenomics, which enhanced our understanding of various biological processes (Misra et al., 2018). The predictions by integrating genomic and metabolomic data commonly generate better results than the predictions by single-omics or other combined omic data in rice (Wang S. et al., 2019). The need for and the significance of the integration of multi-omics has been recognized for plant science research (Jha et al., 2017; Zhao et al., 2021). To combat the increasing challenges of climate change on crops, integrating the multiomics method to study plant science is required (Jha et al., 2017). Designing, conducting, and interpreting multiomics studies can help us to understand plant biology with multiple layers of information on biological data (Haas et al., 2017). Multi-omics can facilitate technique-assisted breeding, genetic engineering, and genome editing and thereby accelerate crop improvement. Existed insights regarding plant response to abiotic stresses by integrating omics are shown in Table 1, which was expressed in detail as follows.

**TABLE 1:** Integration of omics study in crops responding to abiotic stresses.

Species	Applied omics	Growth stage	Sample	Stress type, duration and intensity	References
Soybean	Transcriptomics, proteomics	Seedling stage	Roots	Heat stress, 40°C for 24 h	Valdés-López et al., 2016
Rice	Transcriptomics, proteomics	Seedling stage	Leaves	Quinclorac stress, 0.1 mM quinclorac herbicide for 6 h	Wang et al., 2017
Cotton	Transcriptomics, proteomics	Seedling stage	Leaves	Salt stress, 200 mM NaCl for 4 or 24 h	Peng et al., 2018
Rice	Transcriptomics, proteomics	Seedling stage	Roots	Drought stress, decreased water supply for 4 day	Anupama et al., 2019
Maize	Transcriptomics, proteomics	Seedling stage	Leaves	Zinc deficiency, no Zn supply for 10 and 15 day	Zhang et al., 2019
Hulless barley	Transcriptomics, proteomics	Germination	Seeds	Salt stress, 200 mM NaCl for 4 and 16 h	Lai et al., 2020
Rapeseed	Transcriptomics, proteomics	Seedling stage	Leaves	Cold stress, 8°C/4°C (day/night) for 7 day	Mehmood et al., 2021
Rapeseed	Transcriptomics, proteomics	Seedling stage	Leaves	Freezing stress, −4°C for 12 h	Wei et al., 2021
Rice	Transcriptomics, metabolomics	Flowering stage	Anthers, pistils before pollination and pollinated pistils	Heat stress, 38°C for 6 h; drought stress, withdraw water for 5 day; combined heat and drought	Li et al., 2015
Pepper	Transcriptomics, metabolomics	Seedling stage	Leaves	Heat stress, 40° for 28 h	Wang J. et al., 2019
Wheat	Transcriptomics, metabolomics	Seedling stage	Crowns	Cold acclimation, 4° for 28 day; freezing stress, −5°C for 24 h	Zhao et al., 2019
Rapeseed	Transcriptomics, metabolomics	Seedling stage	Leaves	Cold stress, 4°C for 12 h	Jian et al., 2020
Foxtail millet	Transcriptomics, metabolomics	Seedling stage	Roots	Salt stress, 150 mM NaCl for 7 day	Pan et al., 2020
Tomato	Transcriptomics, metabolomics	Seedling stage	Leaves and roots	Salt stress, 200 mM NaCl for 7 day	Mellidou et al., 2021
Canola	Transcriptomics, metabolomics	Seedling stage	Roots	Alkaline salt stress, 40 mm of Na_2_CO_3_ for 3 day	Wang et al., 2021
Soybean	Transcriptomics, metabolomics	Seedling stage	Whole seedlings	Drought stress, 10% PEG for 14 d	Zhao et al., 2021
Wheat	Proteomics, metabolomics	Seedling stage	Leaves	Drought stress, withholding water for 7 day	Michaletti et al., 2018
Wheat	Proteomics, metabolomics	Grain filling stage	Spikes	Heat stress, 37°C for 4 h	Wang et al., 2018
Sugarcane	Proteomics, metabolomics	Vegetative stage	Leaves	Drought stress, 4 and 12 day without irrigation	Budzinski et al., 2019
Rice	Proteomics, metabolomics	Three-months plants	Spikes	Abrupt drought-flood alternation stress, naturally dried for 10 day and then submerged in water-filled box for 8 day	Xiong et al., 2019
Rice	Proteomics, metabolomics	Seedling stage	Leaves	Drought stress, withdraw water for 2 day	Du et al., 2020
Soybean	Proteomics, metabolomics	Seedling stage	Leaves	Halosufuron-methyl (HSM) stress, 0.01, 0.05, and 0.5 mg/L HSM for 8 day	Li Y. et al., 2020
Rice	Metabolomics, phenomics	Seedling stage	Roots	Cadmium (Cd) stress, 0.1, 1.0, or 10.0 μM Cd for 3 day	Liu et al., 2021

Integrated analysis of transcriptomics and proteomics was applied to various crops, such as soybean (Valdés-López et al., 2016), rice (Wang et al., 2017; Anupama et al., 2019), maize (Zhang et al., 2019), barley (Lai et al., 2020), and rapeseed (Mehmood et al., 2021; Wei et al., 2021). Valdés-López et al. (2016) found 10 gene regulatory key modules and a variety of proteins that played roles in the heat tolerance of soybean at high temperatures (Valdés-López et al., 2016). Abundant genes and proteins responding to salt stress in hulless barley during seed germination were identified using two contrasting genotypes (Lai et al., 2020). Cold stress induced 48 differentially expressed genes (DEGs) corresponding to 17 differentially expressed proteins (DEPs) in cold-tolerant rapeseed and 82 DEGs corresponding to 38 DEPs in cold-sensitive rapeseed (Mehmood et al., 2021).

The transcriptomics and metabolomics were also integrated to study various crops to different abiotic stresses (Li et al., 2015; Wang J. et al., 2019; Jian et al., 2020; Mellidou et al., 2021; Wang et al., 2021). Sugar metabolism was a key transcriptional and metabolic component that distinguished heat tolerance or susceptibility of the floral organ in rice (Li et al., 2015). High temperature induced a higher accumulation ability of common heat-responsive genes and metabolites in heat-tolerant pepper than in heat-sensitive pepper (Wang J. et al., 2019). Cold acclimation and freezing induced the reaction of proline-synthesis and abscisic acid (ABA) and jasmonic acid (JA)-Ile signal transduction pathways in wheat (Zhao et al., 2019). Pan et al. (2020) suggested that phenylpropanoid, flavonoid, and lignin biosynthesis pathways and lysophospholipids played roles in the salinity tolerance of foxtail millet (Pan et al., 2020). Recently, Zhao et al. (2021) generated a regulatory mechanism for flavonoid secondary metabolism and adaptive characteristics of soybean under drought stress (Zhao et al., 2021).

A combination of proteomics and metabolomics revealed a regulatory network in crops to abiotic stresses, such as heat (Wang et al., 2018), drought (Michaletti et al., 2018; Budzinski et al., 2019; Du et al., 2020), and drought–flood alternation (Xiong et al., 2019). Michaletti et al. (2018) revealed that total protein content decreased more severely in drought-sensitive wheat than in drought-tolerant wheat and drought stress changed the abundance of main metabolites (Michaletti et al., 2018). Proteomic and metabolomic analyses identified 309 heat-responsive proteins and 98 metabolites specifically altered in wheat at high temperatures, respectively (Wang et al., 2018). High storage of protein content and high stable filling rate in wheat at heat stress are due to the reallocation of energy to heat protection and reserves deposition (Wang et al., 2018). Du et al. (2020) found that the drought response of rice was altered by nitrogen management methods as indicated by differentially expressed proteins and differential metabolites (Du et al., 2020). Halosufuron-methyl (HSM) stress induced the accumulation of detoxification-related proteins and metabolites in soybean (Li Y. et al., 2020). Furthermore, integration analysis of metabolomics and phenomics found that multiple strategies were employed to increase the tolerance of rice root to cadmium stress, such as upregulation of lipids, fatty acids, and phenylethanoid glycosides (Liu et al., 2021).

With the rapid development of high-throughput omics methods, it is quite challenging to interpret the multidimensional data from genomics, transcriptomics, proteomics, metabolomics, and phenomics and translate the data into a biologically meaningful context (Misra et al., 2018). Even though the individual omic data might not be “big data,” their integration could turn them to “big data.” To better overcome the difficulties, putting more effort into experimental design, such as plant cultivation, sample collection, and extraction, was necessary as the first step. The normalization, transformation, and scaling of the individual omic data should take the integration of multi-omics into account. More importantly, forming a culture of multiomics data sharing and multidisciplinary collaboration can support us to conduct integrated analysis across disciplines and better investigate tolerance characteristics.

CRISPR/Cas genome editing has emerged as a revolutionary technique for making accurate, robust, and efficient genetic manipulations in plant genomes, especially for engineering abiotic stress tolerance of crops (Zafar et al., 2020). Klap et al. (2017) confirmed that SlAGAMOUS-LIKE 6 (SIAGL6) played roles in tomato fruit settings under heat stress and was responsible for the parthenocarpic phenotype by combining marker-assisted mapping, the next-generation sequencing with CRISPR/Cas9 gene knockout (Klap et al., 2017). The integration of multi-omics can benefit the extensive application of CRISPR/Cas9 technology in crop improvement to climate challenges.

## Conclusion

The response of crops to various stresses is considered a complicated process. Integrated techniques and strategies of omics in stress-tolerance study will provide new insights for crop improvement at different levels. Constructing regulatory mechanisms hubs by integrating the omics method can help us to understand systematically the entire behavior and adaption of crops to abiotic stresses. The development of new crop varieties using modern multiomics technologies will help us to combat the emerging challenges of climate change effects on crops and meet the high demand for population growth in the next decade. Here, we suggested that the new emerging phenomics should be integrated more with other omics, especially for screening heat-tolerant crops. The function of key genes responsible for heat tolerance in the levels of metabolites, proteins, and phenotypes can be further validated with the new-emerging CRISPR/Cas technique. Furthermore, several stress conditions can occur concurrently, especially in the field. Tolerance of crops to single stress might not correspond to that to combined stress. Thereby, more efforts are required in the future in the response of crops to combined stress using multi-omics. Meanwhile, most of the studies now focused on the response of roots or leaves in crops, while the response of reproductive organs in crops was more important to study since it was directly associated with crop yield. In order to uncover new and breakthrough insights in stress resilience and tolerance of crops using multiomics techniques, multidisciplinary collaborations from plant scientists and computer scientists, breeding companies, and the farmer should create a knowledge sharing and joint community.

## Author Contributions

RZ wrote the draft. FJ and LN collected the references. XS, LY, YY, and ZW gave valuable comments on the manuscript. All authors contributed to the article and approved the submitted version.

## Conflict of Interest

The authors declare that the research was conducted in the absence of any commercial or financial relationships that could be construed as a potential conflict of interest.

## Publisher’s Note

All claims expressed in this article are solely those of the authors and do not necessarily represent those of their affiliated organizations, or those of the publisher, the editors and the reviewers. Any product that may be evaluated in this article, or claim that may be made by its manufacturer, is not guaranteed or endorsed by the publisher.

## References

[B1] Anupama A. Bhugra S. Lall B. Chaudhury S. Chugh A. (2019). Morphological, transcriptomic and proteomic responses of contrasting rice genotypes towards drought stress. *Environ. Exp. Bot*. 166:103795. 10.1016/j.envexpbot.2019.06.008

[B2] Bhatta M. Morgounov A. Belamkar V. Baenziger P. S. (2018). Genome-wide association study reveals novel genomic regions for grain yield and yield-related traits in drought-stressed synthetic hexaploid wheat. *Int. J. Mol. Sci*. 19:3011. 10.3390/ijms19103011 30279375 PMC6212811

[B3] Bohnert H. J. Gong Q. Li P. Ma S. (2006). Unraveling abiotic stress tolerance mechanisms–getting genomics going. *Curr. Opin. Plant Biol.* 9 180–188. 10.1016/j.pbi.2006.01.003 16458043

[B4] Budzinski I. Moraes F. Cataldi T. R. Franceschini L. M. Labate C. A. (2019). Network analyses and data integration of proteomics and metabolomics from leaves of two contrasting varieties of sugarcane in response to drought. *Front. Plant Sci*. 10:1524. 10.3389/fpls.2019.01524 31850025 PMC6892781

[B5] Chaudhary S. Devi P. Bhardwaj A. Jha U. C. Sharma K. D. Prasad P. V. (2020). Identification and characterization of contrasting genotypes/cultivars for developing heat tolerance in agricultural crops: current status and prospects. *Front. Plant Sci*. 11:587264. 10.3389/fpls.2020.587264 33193540 PMC7642017

[B6] Chebrolu K. K. Fritschi F. B. Ye S. Krishnan H. B. Smith J. R. Gillman J. D. (2016). Impact of heat stress during seed development on soybean seed metabolome. *Metabolomics* 12:28.

[B7] Chen J. Chopra R. Hayes C. Morris G. Burow G. (2017). Genome-wide association study of heat tolerance of developing leaves during vegetative growth stages in a sorghum association panel. *Plant Genom*. 10 1–15. 10.3835/plantgenome2016.09.0091 28724078

[B8] Das A. Rushton P. J. Rohila J. S. (2017). Metabolomic profiling of soybeans (*Glycine max* L.) reveals the importance of sugar and nitrogen metabolism under drought and heat stress. *Plants* 6:21. 10.3390/plants6020021 28587097 PMC5489793

[B9] Devireddy A. R. Zandalinas S. I. Fichman Y. Mittler R. (2021). Integration of reactive oxygen species and hormone signaling during abiotic stress. *Plant J*. 105 459–476. 10.1111/tpj.15010 33015917

[B10] Du J. Shen T. Xiong Q. Zhu C. Peng X. He X. (2020). Combined proteomics, metabolomics and physiological analyses of rice growth and grain yield with heavy nitrogen application before and after drought. *BMC Plant Biol*. 20:556. 10.1186/s12870-020-02772-y 33302870 PMC7731554

[B11] Egea-Cortines M. Doonan J. H. (2018). Editorial: phenomics. *Front. Plant Sci*. 9:678. 10.3389/fpls.2018.00678 29875783 PMC5974546

[B12] Fichman Y. Miller G. Mittler R. (2019). Whole-plant live imaging of reactive oxygen species. *Mol. Plant*. 12 1203–1210. 10.1016/j.molp.2019.06.003 31220601

[B13] Furbank R. T. Tester M. (2011). Phenomics – technologies to relieve the phenotyping bottleneck. *Trends Plant Sci*. 16 635–644. 10.1016/j.tplants.2011.09.005 22074787

[B14] González-Schain N. Dreni L. Lawas L. M. Galbiati M. Colombo L. Heuer S. (2016). Genome-wide transcriptome analysis during anthesis reveals new insights into the molecular basis of heat stress responses in tolerant and sensitive rice varieties. *Plant Cell Physiol*. 57 57–68. 10.1093/pcp/pcv174 26561535

[B15] Haas R. Zelezniak A. Iacovacci J. Kamrad S. Townsend S. Ralser M. (2017). Designing and interpreting ‘multi-omic’ experiments that may change our understanding of biology. *Curr. Opin. Syst. Biol*. 6 37–45. 10.1016/j.coisb.2017.08.009 32923746 PMC7477987

[B16] Hakeem K. R. Chandna R. Ahmad P. Iqbal M. Ozturk M. (2012). Relevance of proteomic investigations in plant abiotic stress physiology. *Omics* 16 621–635. 10.1089/omi.2012.0041 23046473

[B17] He X. Guo S. Wang Y. Wang L. Sun S. Sun J. (2020). Systematic identification and analysis of heat-stress-responsive lncRNAs, circRNAs and miRNAs with associated co-expression and ceRNA networks in cucumber (*Cucumis sativus* L.). *Physiol. Planta*. 168 736–754. 10.1111/ppl.12997 31125116

[B18] Hickey L. T. Hafeez A. N. Robinson H. Jackson S. A. Leal-Bertioli S. C. Tester M. (2019). Breeding crops to feed 10 billion. *Nat. Biotechnol.* 37 744–754. 10.1038/s41587-019-0152-9 31209375

[B19] Hu P. Zheng Q. Luo Q. Teng W. Li H. Li B. (2021). Genome-wide association study of yield and related traits in common wheat under salt-stress conditions. *BMC Plant Biol*. 21:27. 10.1186/s12870-020-02799-1 33413113 PMC7792188

[B20] Janni M. Gullì M. Maestri E. Marmiroli M. Valliyodan B. Nguyen H. T. (2020). Molecular and genetic bases of heat stress responses in crop plants and breeding for increased resilience and productivity. *J. Exp. Bot.* 71 3780–3802. 10.1093/jxb/eraa034 31970395 PMC7316970

[B21] Jean-Baptiste K. McFaline-Figueroa J. L. Alexandre C. M. Dorrity M. W. Saunders L. Bubb K. L. (2019). Dynamics of gene expression in single root cells of *Arabidopsis thaliana*. *Plant Cell* 31 993–1011. 10.1105/tpc.18.00785 30923229 PMC8516002

[B22] Jha U. C. Bohra A. Parida S. K. Jha R. (2017). Integrated “omics” approaches to sustain global productivity of major grain legumes under heat stress. *Plant Breed*. 136 437–459. 10.1111/pbr.12489

[B23] Jha U. C. Nayyar H. Palakurthi R. Jha R. Thudi M. (2021). Major QTLs and potential candidate genes for heat stress tolerance identified in chickpea (*Cicer arietinum* L.). *Front Plant Sci*. 12:655103. 10.3389/fpls.2021.655103 34381469 PMC8350164

[B24] Jian H. Xie L. Wang Y. Cao Y. Wan M. Lv D. (2020). Characterization of cold stress responses in different rapeseed ecotypes based on metabolomics and transcriptomics analyses. *PeerJ* 8:e8704. 10.7717/peerj.8704 32266113 PMC7120054

[B25] Karwa S. Bahuguna R. N. Chaturvedi A. K. Maurya S. Arya S. S. Chinnusamy V. (2020). Phenotyping and characterization of heat stress tolerance at reproductive stage in rice (*Oryza sativa* L.). *Acta Physiol. Plant* 42:29.

[B26] Kersey P. J. (2019). Plant genome sequences: past, present, future. *Curr. Opin. Plant Biol.* 48 1–8. 10.1016/j.pbi.2018.11.001 30579050

[B27] Khan Z. Shahwar D. (2020). “Role of heat shock proteins (HSPs) and heat stress tolerance in crop plants,” in *Sustainable Agriculture in the Era of Climate Change*, eds Roychowdhury R. Choudhury S. Hasanuzzaman M. Srivastava S. (Cham: Springer), 211–234. 10.1007/978-3-030-45669-6_9

[B28] Kilasi N. L. Singh J. Vallejos C. E. Ye C. Jagadish S. V. Kusolwa P. (2018). Heat stress tolerance in rice (*Oryza sativa* L.): identification of quantitative trait loci and candidate genes for seedling growth under heat stress. *Front. Plant Sci*. 9:1578. 10.3389/fpls.2018.01578 30443261 PMC6221968

[B29] Klap C. Yeshayahou E. Bolger A. M. Arazi T. Gupta S. K. Shabtai S. (2017). Tomato facultative parthenocarpy results from Sl AGAMOUS-LIKE 6 loss of function. *Plant Biotechnol. J.* 15 634–647. 10.1111/pbi.12662 27862876 PMC5399002

[B30] Lai X. Bazin J. Webb S. Crespi M. Zubieta C. Conn S. J. (2018). “Circular RNAs in Plants”. in *Circular RNAs, Advances in Experimental Medicine and Biology*, ed. Xiao J. (Singapore: Springer), 329–343. 10.1007/978-981-13-1426-1_26 30259378

[B31] Lai Y. Zhang D. Wang J. Wang J. Wang H. (2020). Integrative transcriptomic and proteomic analyses of molecular mechanism responding to salt stress during seed germination in hulless barley. *Int. J. Mol. Sci*. 21:359. 10.3390/ijms21010359 31935789 PMC6981547

[B32] Li J. Cui J. Dai C. Liu T. Cheng D. Luo C. (2020). Whole-transcriptome RNA sequencing reveals the global molecular responses and ceRNA regulatory network of mRNAs, IncRNAs, miRNAs and circRNAs in response to salt stress in sugar beet (*Beta vulgaris*). *Int. J. Mol. Sci*. 22:289. 10.3390/ijms22010289 33396637 PMC7795855

[B33] Li X. Lawas L. M. Malo R. Glaubitz U. Erban A. Mauleon R. (2015). Metabolic and transcriptomic signatures of rice floral organs reveal sugar starvation as a factor in reproductive failure under heat and drought stress. *Plant Cell Environ*. 38 2171–2192. 10.1111/pce.12545 25828772

[B34] Li X. Zilong G. Lv Y. Cen X. Ding X. Wu H. (2017). Genetic control of the root system in rice under normal and drought stress conditions by genome-wide association study. *PLoS Genet*. 13:e1006889. 10.1371/journal.pgen.1006889 28686596 PMC5521850

[B35] Li Y. Xiao J. Chen L. Huang X. Cheng Z. Han B. (2018). Rice functional genomics research: past decade and future. *Mol. Plant* 11 359–380. 10.1016/j.molp.2018.01.007 29409893

[B36] Li Y. Zhang Q. Yu Y. Li X. Tan H. (2020). Integrated proteomics, metabolomics and physiological analyses for dissecting the toxic effects of halosulfuron-methyl on soybean seedlings (*Glycine max* merr.). *Plant Physiol. Bioch*. 157 303–315. 10.1016/j.plaphy.2020.10.033 33157422

[B37] Liao J. L. Zhou H. W. Peng Q. Zhong P. A. Zhang H. Y. He C. (2015). Transcriptome changes in rice (*Oryza sativa* L.) in response to high night temperature stress at the early milky stage. *BMC Genom*. 16:18. 10.1186/s12864-015-1222-0 25928563 PMC4369907

[B38] Liu C. Lan M. M. He E. K. Yao A. J. Wang G. B. Tang Y. T. (2021). Phenomic and metabolomic responses of roots to cadmium reveal contrasting resistance strategies in two rice cultivars (*Oryza sativa* L.). *Soil Ecol. Lett*. 3 220–229. 10.1007/s42832-021-0088-0

[B39] Lu Y. Li R. Wang R. Wang X. Zheng W. Sun Q. (2017). Comparative proteomic analysis of flag leaves reveals new insight into wheat heat adaptation. *Front. Plant Sci*. 8:1086. 10.3389/fpls.2017.01086 28676819 PMC5476934

[B40] Ma Y. Qin F. Tran L. P. (2012). Contribution of genomics to gene discovery in plant abiotic stress responses. *Mol. Plant* 5 1176–1178. 10.1093/mp/sss085 22930735

[B41] Mangrauthia S. K. Bhogireddy S. Agarwal S. Prasanth V. V. Voleti S. R. Neelamraju S. (2017). Genome-wide changes in microRNA expression during short and prolonged heat stress and recovery in contrasting rice cultivars. *J. Exp. Bot*. 68 2399–2412. 10.1093/jxb/erx111 28407080 PMC5447883

[B42] Mazzeo M. F. Cacace G. Iovieno P. Massarelli I. Grillo S. Siciliano R. A. (2018). Response mechanisms induced by exposure to high temperature in anthers from thermo-tolerant and thermo-sensitive tomato plants: a proteomic perspective. *PLoS One* 13:e0201027. 10.1371/journal.pone.0201027 30024987 PMC6053223

[B43] Mehmood S. S. Lu G. Luo D. Hussain M. A. Raza A. Zafar Z. (2021). Integrated analysis of transcriptomics and proteomics provides insights into the molecular regulation of cold response in *Brassica napus*. *Environ. Exp. Bot*. 187:104480. 10.1016/j.envexpbot.2021.104480

[B44] Mellidou I. Ainalidou A. Papadopoulou A. Leontidou K. Genitsaris S. Karagiannis E. (2021). Comparative transcriptomics and metabolomics reveal an intricate priming mechanism involved in PGPR-mediated salt tolerance in tomato. *Front. Plant Sci.* 12 713984. 10.3389/fpls.2021.713984 34484277 PMC8416046

[B45] Michaletti A. Naghavi M. R. Toorchi M. Zolla L. Rinalducci S. (2018). Metabolomics and proteomics reveal drought-stress responses of leaf tissues from spring-wheat. *Sci. Rep*. 8:5710. 10.1038/s41598-018-24012-y 29632386 PMC5890255

[B46] Misra B. B. Langefeld C. D. Michael O. Cox L. A. (2018). Integrated omics: tools, advances, and future approaches. *J. Mol. Endocrinol.* 61 JME–18–0055. 10.1530/JME-18-0055 30006342

[B47] Mu Q. Zhang W. Zhang Y. Yan H. Liu K. Matsui T. (2017). iTRAQ-based quantitative proteomics analysis on rice anther responding to high temperature. *Int. J. Mol. Sci*. 18:1811. 10.3390/ijms18091811 28832496 PMC5618475

[B48] Ohama N. Sato H. Shinozaki K. Yamaguchi-Shinozaki K. (2017). Transcriptional regulatory network of plant heat stress response. *Trends Plant Sci*. 22 53–65. 10.1016/j.tplants.2016.08.015 27666516

[B49] Orgogozo V. Morizot B. Martin A. (2015). The differential view of genotype–phenotype relationships. *Front. Genet*. 6:179. 10.3389/fgene.2015.00179 26042146 PMC4437230

[B50] Østerberg J. T. Xiang W. Olsen L. I. Edenbrandt A. K. Vedel S. E. Christiansen A. (2017). Accelerating the domestication of new crops: feasibility and approaches. *Trends Plant Sci*. 22 373–384. 10.1016/j.tplants.2017.01.004 28262427

[B51] Pan J. Li Z. Dai S. Ding H. Wang Q. Li X. (2020). Integrative analyses of transcriptomics and metabolomics upon seed germination of foxtail millet in response to salinity. *Sci. Rep*. 10:13660. 10.1038/s41598-020-70520-1 32788682 PMC7423953

[B52] Pandit A. A. Shah R. A. Husaini A. M. (2018). Transcriptomics: a time-efficient tool with wide applications in crop and animal biotechnology. *J. Pharmacogn. Phytochem*. 7 1701–1704.

[B53] Paupière M. J. Müller F. Li H. Rieu I. Tikunov Y. M. Visser R. G. (2017). Untargeted metabolomic analysis of tomato pollen development and heat stress response. *Plant Reprod*. 30 81–94. 10.1007/s00497-017-0301-6 28508929 PMC5486769

[B54] Peng Z. He S. Gong W. Xu F. Pan Z. Jia Y. (2018). Integration of proteomic and transcriptomic profiles reveals multiple levels of genetic regulation of salt tolerance in cotton. *BMC Plant Biol.* 18, 1–19. 10.1186/s12870-018-1350-1 29925319 PMC6011603

[B55] Poudyal D. Rosenqvist E. Ottosen C. O. (2018). Phenotyping from lab to field – tomato lines screened for heat stress using F_*v*_/F_*m*_ maintain high fruit yield during thermal stress in the field. *Funct. Plant Biol*. 46 44–55. 10.1071/FP17317 30939257

[B56] Qi X. Xu W. Zhang J. Guo R. Zhao M. Hu L. (2016). Physiological characteristics and metabolomics of transgenic wheat containing the maize C4 phosphoenolpyruvate carboxylase (PEPC) gene under high temperature stress. *Protoplasma* 254 1017–1030. 10.1007/s00709-016-1010-y 27491550

[B57] Rascio A. Santis G. D. Sorrentino G. (2020). A low-cost method for phenotyping wilting and recovery of wheat leaves under heat stress using semi-automated image analysis. *Plants* 9:718. 10.3390/plants9060718 32516905 PMC7355443

[B58] Razzaq A. Sadia B. Raza A. Hameed M. K. Saleem F. (2019). Metabolomics: a way forward for crop improvement. *Metabolites* 9:303. 10.3390/metabo9120303 31847393 PMC6969922

[B59] Rich-Griffin C. Stechemesser A. Finch J. Lucas E. Ott S. Schfer P. (2020). Single-Cell transcriptomics: A high-resolution avenue for plant functional genomics. *Trends Plant Sci*. 25 186–197. 10.1016/j.tplants.2019.10.008 31780334

[B60] Rosenqvist E. Großkinsky D. K. Ottosen C. O. van de Zedde R. (2019). The Phenotyping Dilemma—The challenges of a Diversified Phenotyping Community. *Front. Plant Sci*. 10:163. 10.3389/fpls.2019.00163 30873188 PMC6403123

[B61] Schiessl S. V. Quezada-Martinez D. Orantes-Bonilla M. Snowdon R. J. (2020). Transcriptomics reveal high regulatory diversity of drought tolerance strategies in a biennial oil crop. *Plant Sci*. 297:110515. 10.1016/j.plantsci.2020.110515 32563455

[B62] Shakoor N. Lee S. Mockler T. (2017). High throughput phenotyping to accelerate crop breeding and monitoring of diseases in the field. *Curr. Opin. Plant Biol*. 38 184–192. 10.1016/j.pbi.2017.05.006 28738313

[B63] Sharma D. K. Andersen S. B. Ottosen C. O. Rosenqvist E. (2012). Phenotyping of wheat cultivars for heat tolerance using chlorophyll a fluorescence. *Funct. Plant Biol*. 39 936–947. 10.1071/FP12100 32480843

[B64] Sharma D. K. Torp A. M. Rosenqvist E. Ottosen C. O. Andersen S. B. (2017). QTLs and potential candidate genes for heat stress tolerance identified from the mapping populations specifically segregating for F_*v*_/F_*m*_ in wheat. *Front. Plant Sci*. 8:1668. 10.3389/fpls.2017.01668 29021798 PMC5623722

[B65] Shi F. Xu H. Liu C. Tan C. Ren J. Ye X. (2021). Whole-transcriptome sequencing reveals a vernalization-related ceRNA regulatory network in chinese cabbage (*Brassica campestris* L. ssp. *pekinensis*). *BMC Genom*. 22:819. 10.1186/s12864-021-08110-2 34773977 PMC8590779

[B66] Shi J. Yan B. Lou X. Ma H. Ruan S. (2017). Comparative transcriptome analysis reveals the transcriptional alterations in heat-resistant and heat-sensitive sweet maize (*Zea mays* L.) varieties under heat stress. *BMC Plant Biol*. 17:26. 10.1186/s12870-017-0973-y 28122503 PMC5267381

[B67] Shi W. Muthurajan R. Rahman H. Selvam J. Peng S. Zou Y. (2013). Source-sink dynamics and proteomic reprogramming under elevated night temperature and their impact on rice yield and grain quality. *New Phyto*. 197 825–837. 10.1111/nph.12088 23252708

[B68] Sun C. X. Gao X. X. Li M. Q. Fu J. Q. Zhang Y. L. (2016). Plastic responses in the metabolome and functional traits of maize plants to temperature variations. *Plant Biol*. 18 249–261. 10.1111/plb.12378 26280133

[B69] Valdés-López O. Batek J. Gomez-Hernandez N. Nguyen C. T. Isidra-Arellano M. C. Zhang N. (2016). Soybean roots grown under eeat stress show global changes in their transcriptional and proteomic profiles. *Front. Plant Sci*. 7:517. 10.3389/fpls.2016.00517 27200004 PMC4843095

[B70] Wang J. Faisal I. Lan L. Long M. Chong Y. Jin X. (2017). Complementary RNA-sequencing based transcriptomics and iTRAQ proteomics reveal the mechanism of the alleviation of quinclorac stress by salicylic acid in *oryza sativa* ssp. japonica. *Int. J. Mol. Sci*. 18:1975. 10.3390/ijms18091975 28906478 PMC5618624

[B71] Wang J. Lv J. Liu Z. Liu Y. Somg J. Ma Y. (2019). Integration of transcriptomics and metabolomics for pepper (*Capsicum annuum* L.) in response to heat stress. *Int. J. Mol. Sci*. 20:5042. 10.3390/ijms20205042 31614571 PMC6829368

[B72] Wang S. Wei J. Li R. Qu H. Chater J. M. Ma R. (2019). Identification of optimal prediction models using multi-omic data for selecting hybrid rice. *Heredity* 123 395–406. 10.1038/s41437-019-0210-6 30911139 PMC6781126

[B73] Wang W. Pang J. Zhang F. Sun L. Yang L. Zhao Y. (2021). Integrated transcriptomics and metabolomics analysis to characterize alkali stress responses in canola (*Brassica napus* L.). *Plant Physiol. Bioch*. 166 605–620. 10.1016/j.plaphy.2021.06.021 34186284

[B74] Wang X. Dinler B. S. Vignjevic M. Jacobsen S. Wollenweber B. (2015). Physiological and proteome studies of responses to heat stress during grain filling in contrasting wheat cultivars. *Plant Sci*. 230 33–50. 10.1016/j.plantsci.2014.10.009 25480006

[B75] Wang X. Hou L. Lu Y. Wu B. Xue G. Liu M. (2018). Metabolic adaptation of wheat grain contributes to a stable filling rate under heat stress. *J. Exp. Bot*. 69 5531–5545. 10.1093/jxb/ery303 30476278 PMC6255704

[B76] Wei J. Zheng G. Yu X. Liu S. Dong X. Cao X. (2021). Comparative transcriptomics and proteomics analyses of leaves reveals a freezing stress-responsive molecular network in winter rapeseed (*Brassica rapa* L.). *Front. Plant Sci*. 12:664311. 10.3389/fpls.2021.664311 33995460 PMC8113625

[B77] Wen J. Jiang F. Liu M. Zhou R. Sun M. Shi X. (2021). Identification and expression analysis of Cathepsin B-like protease 2 genes in tomato at abiotic stresses especially at High temperature. *Sci. Hortic.* 277:109799. 10.1016/j.scienta.2020.109799

[B78] Wen J. Jiang F. Weng Y. Sun M. Shi X. Zhou Y. (2019). Identification of heat-tolerance QTLs and high-temperature stress-responsive genes through conventional QTL mapping, QTL-seq and RNA-seq in tomato. *BMC Plant Biol*. 19:398–414. 10.1186/s12870-019-2008-3 31510927 PMC6739936

[B79] Werner T. (2010). Next generation sequencing in functional genomics. *Brief Bioinform*. 11 499–511. 20501549 10.1093/bib/bbq018

[B80] Xiong Q. Q. Shen T. H. Zhong L. Zhu C. L. Peng X. S. He X. P. (2019). Comprehensive metabolomic, proteomic and physiological analyses of grain yield reduction in rice under abrupt drought–flood alternation stress. *Physiol. Plant*. 167 564–584. 10.1111/ppl.12901 30561011

[B81] Yang W. Feng H. Zhang X. Zhang J. Doonan J. H. Batchelor W. D. (2020). Crop phenomics and high-throughput phenotyping: past decades, current challenges, and future perspectives. *Mol. Plant* 13 187–214. 10.1016/j.molp.2020.01.008 31981735

[B82] Yang W. Guo Z. Huang C. Duan L. Chen G. Jiang N. (2014). Combining high-throughput phenotyping and genome-wide association studies to reveal natural genetic variation in rice. *Nat. Commun*. 5:5087. 10.1038/ncomms6087 25295980 PMC4214417

[B83] Zafar S. A. Zaidi S. S. E. A. Gaba Y. Singla-Pareek S. L. Dhankher O. P. Li X. (2020). Engineering abiotic stress tolerance via CRISPR/Cas-mediated genome editing. *J. Exp. Bot.* 71 470–479. 10.1093/jxb/erz476 31644801

[B84] Zhang H. Y. Lei G. Zhou H. W. He C. Liao J. L. Huang Y. J. (2017). Quantitative iTRAQ-based proteomic analysis of rice grains to assess high night temperature stress. *Proteomics* 17:1600365. 10.1002/pmic.201600365 28101936 PMC5811895

[B85] Zhang J. Wang S. Song S. Xu F. Pan Y. Wang H. (2019). Transcriptomic and proteomic analyses reveal new insight into chlorophyll synthesis and chloroplast structure of maize leaves under zinc deficiency stress. *J. Proteom*. 199 123–134. 10.1016/j.jprot.2019.03.001 30849524

[B86] Zhao B. Zhang S. Yang W. Li B. Zhang X. (2021). Multi-omic dissection of the drought resistance traits of soybean landrace LX. *Plant Cell Environ*. 44 1379–1398. 10.1111/pce.14025 33554357

[B87] Zhao F. Zhang D. Zhao Y. Wang W. Yang H. Tai F. (2016). The difference of physiological and proteomic changes in maize leaves adaptation to drought, heat, and combined both stresses. *Front. Plant Sci*. 7:1471. 10.3389/fpls.2016.01471 27833614 PMC5080359

[B88] Zhao Y. Zhou M. Xu K. Li J. Li S. Zhang S. (2019). Integrated transcriptomics and metabolomics analyses provide insights into cold stress response in wheat. *Crop J*. 7 857–866. 10.1016/j.cj.2019.09.002

[B89] Zhou R. Yu X. Kjær K. H. Rosenqvist E. Ottosen C. O. Wu Z. (2015). Screening and validation of tomato genotypes under heat stress using Fv/Fm to reveal the physiological mechanism of heat tolerance. *Environ. Exp. Bot*. 118 1–11. 10.1016/j.envexpbot.2015.05.006

[B90] Zhou R. Yu X. Ottosen C. O. Zhang T. Wu Z. Zhao T. (2020b). Unique miRNAs and their targets in tomato leaf responding to combined drought and heat stress. *BMC Plant Biol*. 20:107. 10.1186/s12870-020-2313-x 32143575 PMC7060562

[B91] Zhou R. Yu X. Ottosen C. O. Zhao T. (2020a). High throughput sequencing of circRNAs in tomato leaves responding to multiple stresses of drought and heat. *Hortic. Plant J*. 6 34–38. 10.1016/j.hpj.2019.12.004

